# Imaging intercellular biomolecules by using fluorescent protein indicators with lipid-PEG anchors

**DOI:** 10.1038/s41598-026-37240-4

**Published:** 2026-02-02

**Authors:** Marie Mita, Kazuyuki Kiyosue, Tomomi Tani

**Affiliations:** Molecular Biosystems Research Institute, National Institute of Advanced Industrial Science and Technology (AIST), 1-8-31, Midorigaoka, Ikeda, Osaka 563-8577 Japan

**Keywords:** Fluorescent protein (FP)-based indicator, Lipid-PEG anchoring, Extracellular molecules, Glutamate, Potassium ion, Neuronal activity, Biochemistry, Biological techniques, Biophysics, Cell biology, Neuroscience

## Abstract

Extracellular molecules such as ions, amino acids, nucleotides, and proteins are essential mediators of intercellular communication in multicellular organisms. These diffusible factors contribute to development and homeostasis of organisms by coordinating signaling among distant cells. Despite the widespread use of fluorescent indicators for studying intracellular signaling, introducing genetically encoded indicators often complicates the control of their subcellular localization, making it difficult to distinguish intracellular from extracellular signals. Consequently, monitoring the spatiotemporal dynamics of diffusible molecules in the extracellular space has remained technically challenging. Here we report a versatile chemical anchoring method using lipid–polyethylene glycol (PEG) conjugates that immobilizes fluorescent protein (FP)–based indicators on the extracellular surface of living cells. This approach enables stable localization of FP-based indicators on hippocampal neurons in both primary cultures and in acute brain slices. Lipid–PEG–anchored indicator for potassium ion and that for glutamate allowed real-time optical monitoring of ion and neurotransmitter release from neurons during spontaneous and electrically evoked neuronal activity. This non-genetic labeling strategy provides localized, rapidly applicable, high sensitivity, stability, and reproducibility, offering a versatile platform for quantitative monitoring of extracellular signaling events near membranes of living cells in a low invasive manner.

**Supplementary Information:**

The online version contains supplementary material available at 10.1038/s41598-026-37240-4.

## Introduction

Intercellular communication via diffusible molecules is underpinning the coordination of a variety of cellular activities among distant cells during development and maintenance of multicellular organisms. Cells exchange information through membrane permeable molecules and/or membrane-bound ligands, thereby generating spatially and temporally organized signaling networks. Such communication enables multicellular systems to integrate local cellular events into tissue- and organism-level biological events. Among various signaling modalities, diffusible extracellular molecules—including ions, amino acids, nucleotides, and proteins—play critical roles in synchronizing the physiological conditions of distant cells. Despite their importance, the mechanisms by which extracellular signaling molecules propagate, interact, and influence collective cellular behavior remain incompletely understood, largely due to technical limitations in monitoring their rapid diffusion and turnover in extracellular space.

To visualize intercellular interactions and elucidate the information-processing mechanisms of cells, qualitative and quantitative imaging approaches for extracellular molecules have been developed. When tracking the dynamics of interesting molecules, one can directly observe diffusible target molecules with fluorescent tags. However, direct observation of fluorescently tagged diffusible molecules is sometimes challenging due to rapid diffusion that obscures spatial information. This limitation can be overcome by immobilizing a high-affinity binding molecule, which captures the target as it diffuses and thus reports its local distribution. As an example of this approach, the diffusion of Wnt proteins in vivo has been successfully visualized using antibodies immobilized on the extracellular surface of the cell membrane^1,2^. This method enabled, for the first time, to map the spatial distribution and concentration of extracellular signaling molecules. However, the detection of small molecules such as ions and amino acids has remained technically challenging as these molecules are chemically too simple and small to be fluorescently labeled and accommodate direct modification without altering their native properties.

Fluorescent protein (FP)-based indicators, first developed in the 2000s, have emerged as powerful imaging tools with the potential to overcome the challenges of detecting small molecules^3–6^. FP–based indicators report these small molecules by converting binding with these molecules into measurable optical signals, allowing indirect yet highly specific detection of target molecules in their physiological contexts. These indicators typically consist of a fluorescent protein scaffold—such as GFP or its variants—fused to a sensing domain derived from receptors, enzymes, or binding proteins that selectively recognize the target molecule. Upon ligand binding, the conformational change of the sensing domain is transduced into a change in the fluorescence intensity or fluorescence intensity ratio of the fluorescent proteins. This modular architecture enables rational design and optimization of indicators for a wide range of analytes, including ions, nucleotides, amino acids and metabolic molecules, with tunable dynamic ranges and kinetics suited to specific biological questions^7–10^.

Surface-displayed FP-based glutamate indicators, for instance, convert glutamate release into optical signals, enabling visualization of synaptic transmission^7,11–13^. These indicators can be expressed as chimeric proteins containing a transmembrane domain, enabling their display on the extracellular surface of the plasma membrane. This configuration allows them to rapidly detect glutamate released to the extracellular space. However, its practical applications for functionally presenting these indicators on the cell surface are sometimes limited, as the results vary depending on the nature of specific cells and/or indicators. FP-based potassium ion indicators have enabled imaging of intracellular potassium ion dynamics^8,14,15^, but their application to extracellular potassium ion detection remains limited. As a result, extracellular potassium ion has been measured using conventional methods such as microelectrodes or transistor-based sensors, which suffer from drawbacks including limited spatial resolution^16–18^.

To overcome these limitations and monitor the dynamics of extracellular potassium ion, we focused on chemical modification techniques that enable immobilization of molecules onto the extracellular surface of the plasma membrane^19^ and conceived the idea of chemically modifying FP-based indicators. We employed lipid–poly(ethylene glycol) (lipid–PEG) conjugates—PEG derivatives containing lipid chains capable of anchoring biomolecules to the cell membrane^20–22^. By conjugating FP-based glutamate or potassium ion indicators to lipid–PEG, we established a new strategy for visualizing extracellular molecules. When applied to living cells and acute brain slices, these lipid–PEG–anchored indicators enabled highly sensitive and quantitative detection of molecular release induced by neuronal electrical stimulation. Because the indicators are applied extracellularly, there is no contribution from intracellular indicator signals, enabling highly sensitive detection that is strictly confined to the extracellular compartment. Importantly, this chemically anchored method requires no genetic manipulation and can be readily and robustly applied to a wide variety of cell types, ranging from established cell lines to primary cells. More broadly, the lipid–PEG anchoring approach provides a versatile platform for direct visualization and quantification of diffusible extracellular signaling molecules—including neurotransmitters, amino acids, and ions—released from specific cells.

## Results and discussion

### Anchoring fluorescent protein-based potassium ion indicator to the cell surface

We used a fluorescent protein-based potassium ion indicator GINKO2^15^ to observe extracellular potassium ion dynamics. To expose the indicator to the outer surface of the cells, we first employed signal peptides sequences that deliver glutamate indicators to the outer surface of the cells^13^. Specifically, the DNA coding sequence of GINKO2 was inserted between an N-terminal immunoglobulin kappa (Igκ) secretion leader sequence and a C-terminal platelet-derived growth factor receptor (PDGFR) transmembrane domain or a glycosylphosphatidylinositol (GPI) anchor sequence. Fluorescence imaging revealed that GINKO2 fused to either the PDGFR or GPI anchor was transiently expressed in HEK293 cells and localized to the plasma membrane (Supplementary Fig. 1a, b). However, we often observed a substantial amount of fluorescence in cytoplasm as well. Consistent with previous reports^15,23^, the fluorescence signals of these GINKO2 indicators did not respond to changes in extracellular K⁺ concentration (Supplementary Fig. 1c, d). Although the underlying mechanism is unclear, aberrant protein trafficking or misfolding may account for this observation. Consequently, the indicator failed to function at the cell surface.

**Fig. 1:**
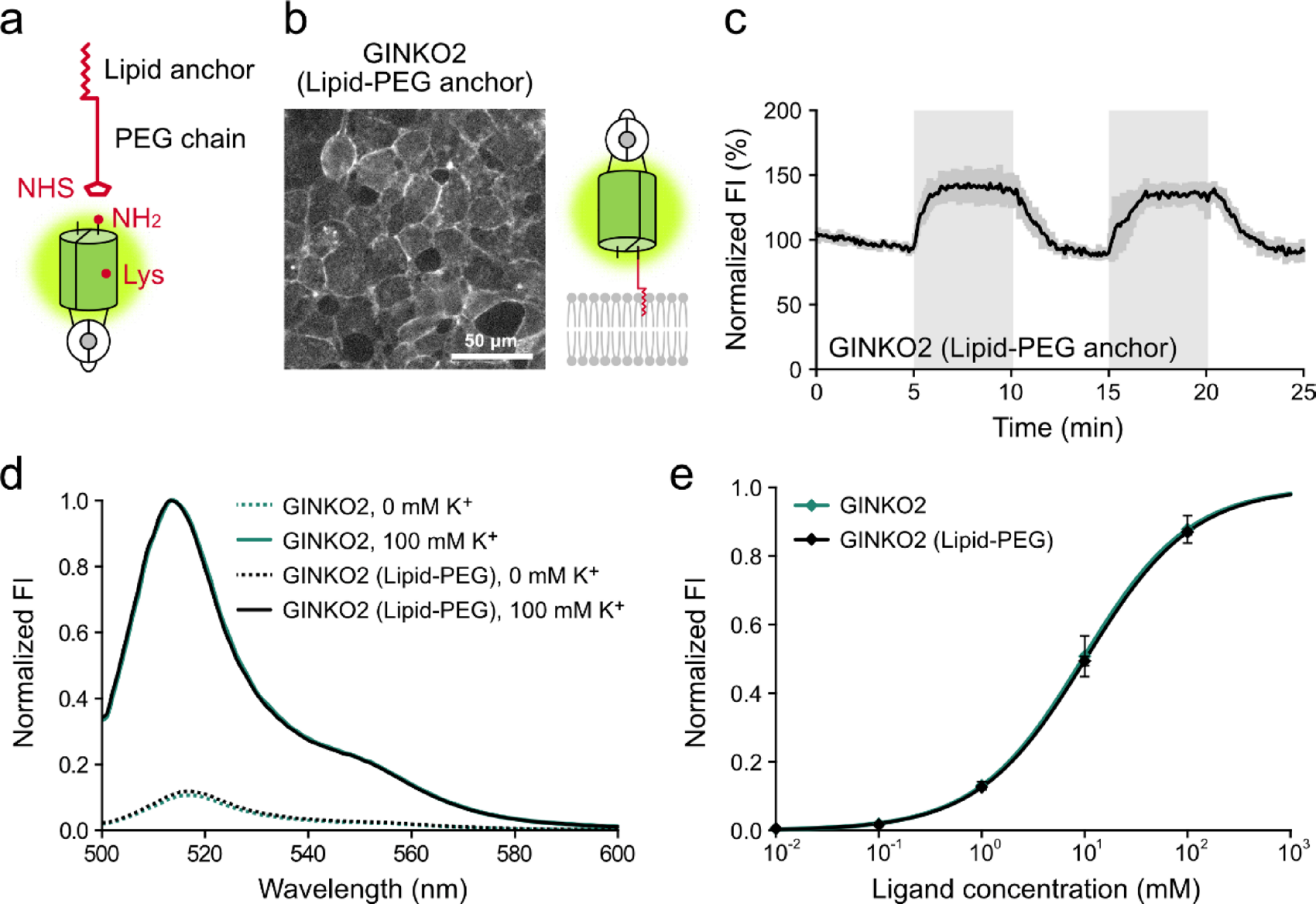
Characterization of lipid-PEG-anchored fluorescent protein-based potassium indicator and live imaging of extracellular glutamate in HEK293 cells. (**a**) Schematic representation of the lipid–PEG derivatives and fluorescent protein–based indicators used in this study. Illustrating the reaction between the NHS ester and the protein N terminus or surface-exposed lysine residues. (**b**) Fluorescence images and schematic representations of fluorescent protein-based potassium ion indicator GINKO2 fused with a chemically introduced lipid-PEG anchor, respectively. Scale: 50 μm. (**c**) Fluorescence intensity (FI) changes in HEK293 cells external loading of lipid-PEG anchored GINKO2. Cells were perfused with solutions containing 3.6 mM K⁺ (baseline) or 50 mM K⁺ using a perfusion device. The solutions containing 50 mM K⁺ was applied during the period indicated with gray backgrounds. FI values were normalized to the average FI over 5 min before the first stimulation (set as 100%). The values represent means ± SD (n = 30 cells from three independent experiments). (**d**) Emission spectra of purified GINKO2 and GINKO2 lipid-PEG in the presence (solid line) or absence (dashed line) of 100 mM K⁺. FI values were normalized to the maximal fluorescence intensity change observed in the presence of K⁺. (**e**) Dose–response curves of purified GINKO2 and GINKO2 lipid-PEG to K⁺. Symbols represent values used for the curve fitting. The curve was fitted with a four-parameter logistic equation, and values represent means ± SD (n = 3) of FI normalized to the upper asymptote.

To overcome this problem, we applied a chemical anchoring method to purified GINKO2 by using lipid-polyethylene glycol (PEG) derivatives^20,24^ (Fig. 1a). The GINKO2 proteins recombinantly expressed and purified with *E. coli* expression system were covalently conjugated to lipid-PEG derivatives (SUNBRIGHT, NOF corporation, Japan). When 1 μM of this conjugate is applied to cultured HEK293 cells, we observed stable and uniform localization of the indicator on the surface membrane of the cells (Fig. 1b). We found that lipid-PEG-anchored GINKO2 showed an increase in fluorescence intensity in response to an addition of K^+^ and returned to baseline levels upon the removal of K^+^ (Fig. 1c, Supplementary video 1).

Importantly, purified lipid-PEG-anchored GINKO2 exhibited decrease in the excitation peak ratio around 400 nm in the presence of K^+^ in in vitro assays (Supplementary Fig. 2); however, the excitation peak near 500 nm, the emission spectrum, and the K^+^ responsiveness were comparable to those observed in purified GINKO2 without lipid-PEG (Fig. 1d, e). These data support that lipid-PEG enables FP-based indicators to be anchored on the extracellular surface of cell membranes without compromising their responsiveness to K^+^, allowing them to image the dynamics of extracellular K^+^.

The N-hydroxysuccinimide (NHS) ester at the terminus of the lipid–PEG derivative reacts non-specifically with primary amines, including the N-terminus and surface-exposed lysine residues of proteins, to form stable amide bonds. Consequently, conjugation can occur not only at the N-terminus of the protein but also at multiple sites on the protein surface. Although targeting specific lysine residues on the surface could, in principle, provide greater control over the conjugation site, such modifications may substantially alter the intrinsic properties of the FP-based indicator. While in vitro assays revealed no major alterations in the fundamental properties of the GINKO2 upon lipid–PEG conjugation, differences in indicator orientation on the cell surface could potentially influence ligand detection in cellular contexts.

### Imaging spontaneous glutamate release from primary cultured hippocampus neurons with lipid-PEG anchored indicators

**Fig. 2:**
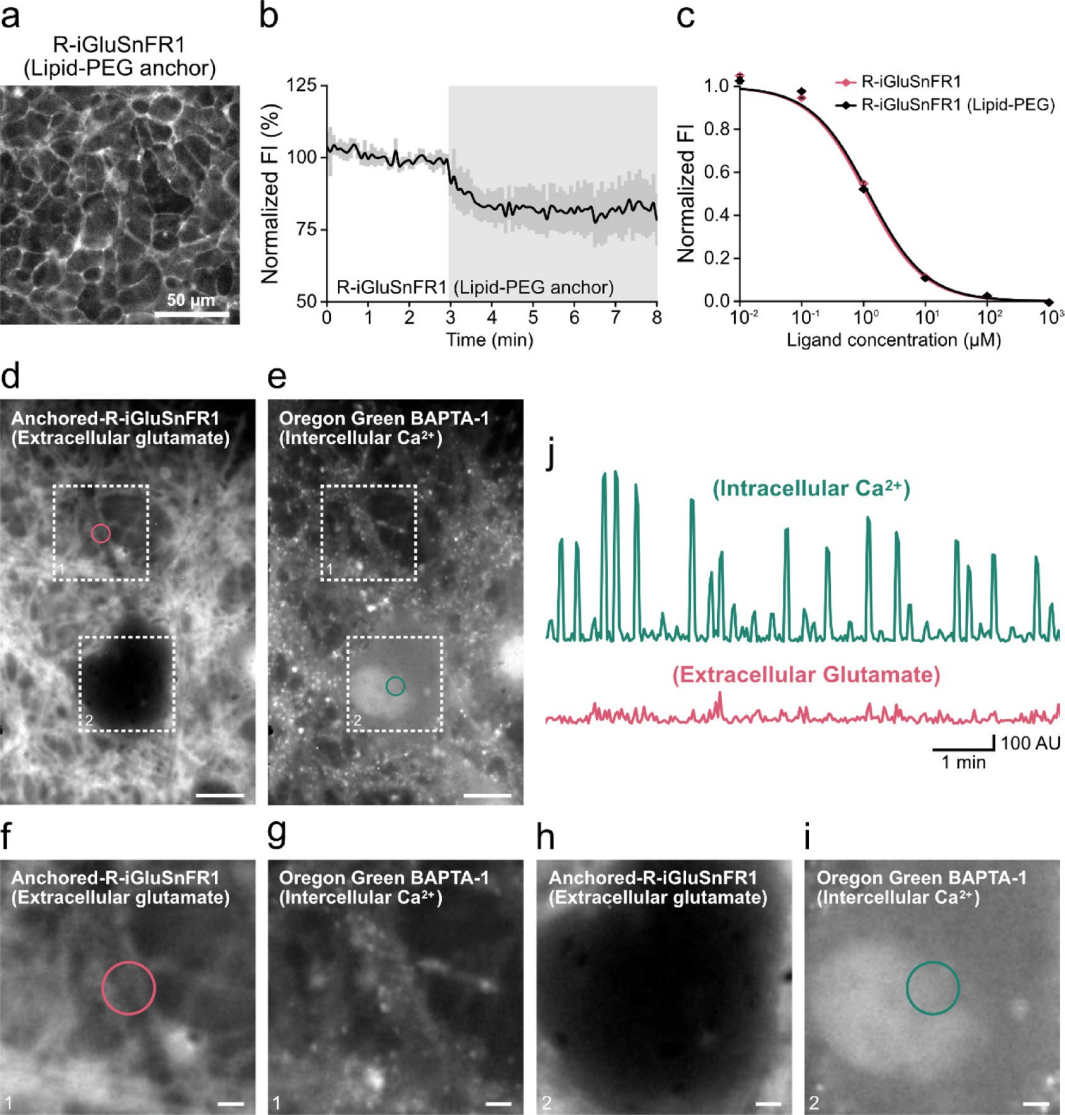
Characterization of lipid-PEG-anchored fluorescent protein–based glutamate indicator and live imaging of extracellular glutamate in HEK293 cells and primary cultured cells. (**a**) Fluorescence images of R-iGluSnFR1 fused with a chemically introduced lipid-PEG anchor in HEK293 cells. Scale: 50 μm. (**b**) Fluorescence intensity (FI) changes in HEK293 cells external loading of lipid-PEG anchored R-iGluSnFR1 (Lipid-PEG anchor). Cells were perfused with solutions containing 0 mM (baseline) or 1 mM glutamate using a perfusion device. The solutions containing 1 mM glutamate was applied during the period indicated with gray backgrounds. FI values were normalized to the average FI over 3 min before the first stimulation (set as 100%). The values represent means ± SD (n = 30 cells from three independent experiments). (**c**) Dose–response curves of purified R-iGluSnFR1 and R-iGluSnFR1 lipid-PEG to glutamate. Symbols represent values used for the curve fitting. The curve was fitted with a four-parameter logistic equation, and values represent means ± SD (n = 3) of FI normalized to the upper asymptote. (**d–i**) Fluorescence images and magnified images of primary cultured mouse hippocampal neurons co-loaded with lipid–PEG–anchored R-iGluSnFR1 (**d, f, h**) and Oregon Green BAPTA-1 (**e, g, i**). Areas indicated by circles represent the regions for the fluorescence intensity measurements. Scales: 10 μm and 2 μm, respectively. (**j**) Time courses of fluorescence intensity changes of lipid-PEG-anchored R-iGluSnFR1 and Oregon Green BAPTA-1 during dual imaging in primary cultured mouse hippocampal neurons. Plotted values represent the absolute difference in fluorescence intensity between each frame and the preceding frame.

To examine whether this anchoring method could be applied to FP-based indicators other than the potassium ion indicator, we performed imaging using the FP-based glutamate indicator, R-iGluSnFR1^12^. R-iGluSnFR1 can be expressed as a chimeric protein fused to a PDGFR-derived membrane anchor and has been reported to function at the extracellular surface. When HEK293 cells were treated with lipid-PEG-anchored R-iGluSnFR1, fluorescence was observed on the outer surface of the cells (Fig. 2a, b). This anchoring method was also applied to the FP-based glutamate indicator R-iGluSnFR1 without affecting its responsiveness to glutamate (Fig. 2c). These results demonstrate that the lipid-PEG anchoring approach can be successfully applied to the FP-based glutamate indicator R-iGluSnFR1 without affecting its glutamate responsiveness, allowing visualization of extracellular glutamate distribution in live-cell imaging.

R-iGluSnFR1 displayed on the plasma membrane has also been reported to detect electrically evoked glutamate release in primary cultured mouse hippocampal neurons^12^. To assess whether the lipid–PEG anchoring method can similarly detect neurotransmitter release from neurons, we applied this method to primary cultured neurons. Hippocampal neurons were incubated with the lipid–PEG–anchored R-iGluSnFR1 to detect glutamate release from presynaptic terminals and with the fluorescent Ca^2^⁺ indicator Oregon Green (OG)-BAPTA1 to monitor spontaneous neuronal spiking-activity. We used total internal reflection fluorescence microscopy (TIRFM) for simultaneously imaging these indicators. Changes in red fluorescence signals from lipid–PEG–anchored R-iGluSnFR1 were observed along the contours of cell bodies and neurites (Fig. 2d, f, h). In contrast, fluctuations in green fluorescence signals from OG-BAPTA1 were detected in the cytoplasmic regions of cell bodies and neurites (Fig. 2e, g, i). Together, these findings demonstrate that this dual-color imaging provides a means to simultaneous observation of spontaneous glutamate transients at the cell surface and spiking activities (Fig. 2j), thereby enabling sensitive and qualitative measurements of extracellular neurotransmitter dynamics in neurons without genetic modification.

### Application of lipid-PEG-anchored indicators for imaging extracellular molecules in mouse hippocampal slices

**Fig. 3:**
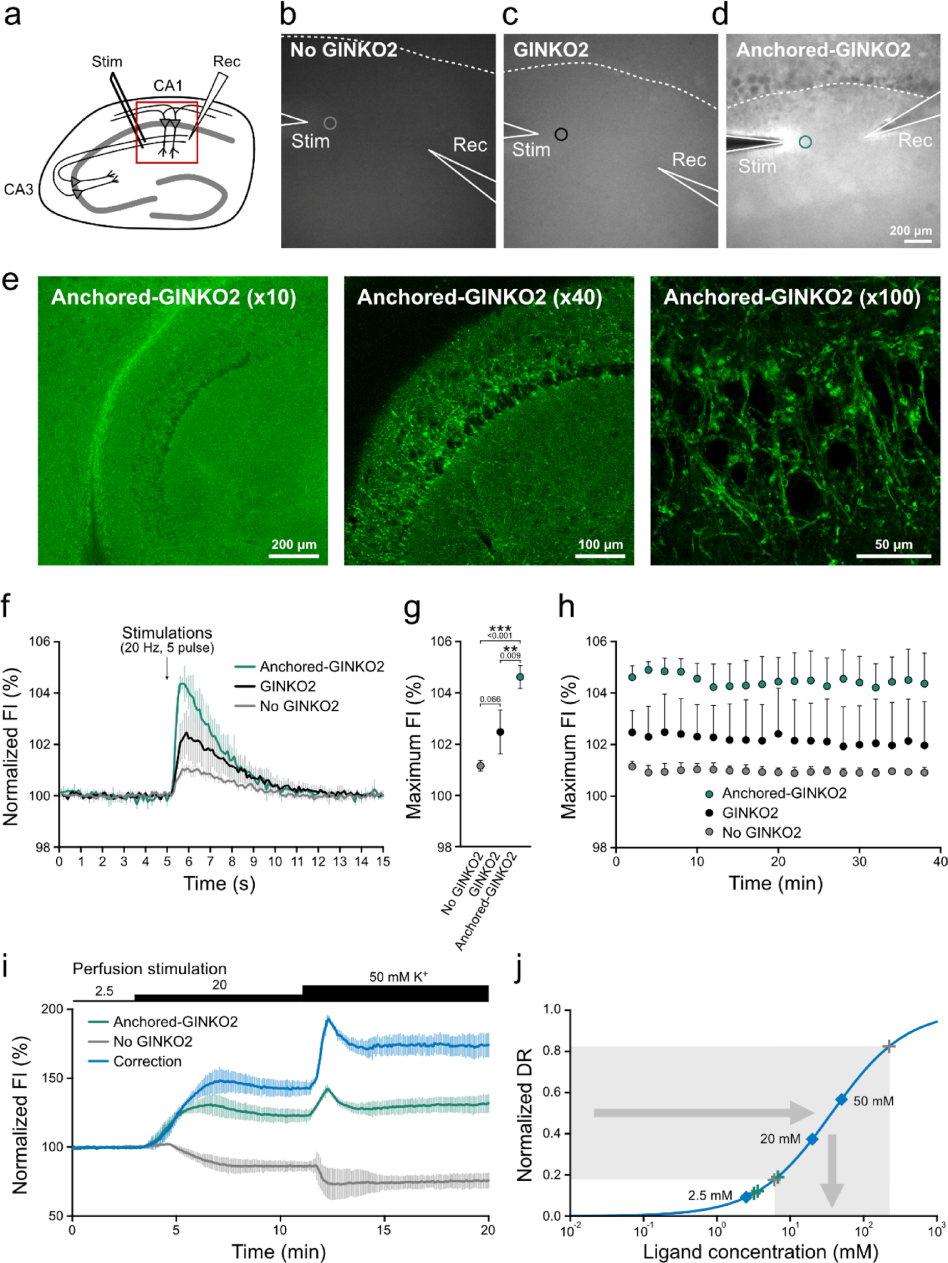
Field stimulation–evoked fluorescence responses in acute hippocampal slices. (**a**) A schematic of mouse hippocampal slice preparation including CA3–CA1 regions. A stimulating electrode and a recording electrode for fEPSP measurement (used in Fig. 4 and subsequent analysis) were shown on the Schaffer collaterals. The red square indicates the CA1 region for fluorescence imaging. (**b-d**) Representative widefield fluorescence images of unstained slices (No GINKO2, **b**), slices loaded with non-anchored GINKO2 (**c**), and lipid-PEG-anchored GINKO2 (**d**). White lines mark electrode positions; dotted lines indicate the CA1 pyramidal cell layer boundary. Areas indicated by circles represent the regions for the fluorescence intensity measurements. Scale, 200 μm. (**e**) Confocal images of lipid-PEG-anchored GINKO2 observed with10 × , 40 × , and 100 × objective lenses. Scales: 200 μm, 100 μm, and 50 μm, respectively. (**f**) Time courses of fluorescence intensity (FI) changes during electrical stimulations (20 Hz, 5 pulse) in unstained (gray, **b**), non-anchored (black, **c**), and anchored slices (green, **d**) in (**b-d**). FI values were normalized to the 5 s pre-stimulation average (set as 100%). Values represent means ± SD (three independent slices). (**g**) Comparison of maximum FI values in (**f**). Statistical significance assessed by one-way ANOVA with Tukey’s post hoc multiple comparison test (∗ ∗ *p* < 0.01; ***p < 0.001). *p*-values are indicated above the SD bars. (**h**) Time-dependent changes in the maximum FI evoked by electrical stimulation in slices under three conditions. Imaging was repeated at 2-min intervals. Values represent mean ± SD (three independent slices). (**i**) Time courses of FI in slices without indicators (gray), with anchored GINKO2 (green) and corrected values (blue). Corrected values were obtained by normalizing the average FI of slices with anchored GINKO2 to that of slices without indicators. Slices were perfused with solutions containing 2.5, 20 or 50 mM K⁺ (indicated by black bars). FI values were normalized to the 3 min pre-stimulation average. Data represent means ± SD (three independent slices). (**j**) Calibration curve generated from corrected FI values in (**i**), fitted using a four-parameter logistic equation. Symbols indicate data used for fitting (blue squares), bend points (gray crosses), and values for K⁺ estimation (green crosses).

To analyze the correlation between neuronal activity and extracellular molecular dynamics, we next evaluated the performance of lipid-PEG–anchored indicators in a different experimental paradigm using acute mouse hippocampal slices. Because synaptic activity can be controlled by electrical stimulation on the Schaffer collateral, the lipid-PEG-anchored GINKO2 has the potential to sense extracellular local potassium dynamics that are elicited by synaptic activities. In brain slices, intrinsic fluorescence signals that reflect redox state of flavoproteins or nicotinamide adenine dinucleotide^25,26^ might affect to the fluorescence imaging of lipid-PEG-anchored GINKO2 as the emission spectrum of GINKO2 overlaps with those of these endogenous fluorophores. To assess the contribution of intrinsic fluorescence to the observed signals, the fluorescence was observed under three conditions: unstained slices, slices loaded with GINKO2 or with lipid-PEG-anchored GINKO2. The tips of the stimulating and recording electrodes were placed in the CA1 stratum radiatum, and fluorescence signals were recorded using a widefield fluorescence microscope (Fig. 3a). Among the three conditions, slices loaded with lipid-PEG-anchored GINKO2 exhibited the highest fluorescence intensity (Fig. 3b-d). In this slice, the dendritic area is stained very brightly, whereas the cell body region appears transparent, suggesting that labeling was restricted to the cell surface (Fig. 3d). To study the spatial distribution of fluorescence in more details, we observed fluorescence images with higher spatial resolution using confocal fluorescence microscopy. Consistent with observations in primary cultured neurons, lipid-PEG-anchored GINKO2 localized along the plasma membrane, confirming membrane-specific labeling in the brain slices as well (Fig. 3e).

We next applied electrical stimulations (20 Hz, 5 pulses) to the Schaffer collateral and monitored changes in fluorescence intensity across the three slice conditions. In all slices, fluorescence transiently increased after the stimulations and returned to the baseline within approximately 10 s (Fig. 3f, Supplementary video 2). When comparing the fluorescence changes following electrical stimulations, slices treated with lipid-PEG-anchored GINKO2 exhibited significantly higher responses than unstained slices, whereas slices treated with non-anchored GINKO2 showed negligible changes (Fig. 3g). These results indicate that lipid-PEG-anchored fluorescent indicators enable reliable monitoring of extracellular molecular dynamics in the brain slice preparations.

Importantly, during the continuous 40 min observations, slices loaded with lipid-PEG-anchored GINKO2 maintained stable fluorescence responses, with consistent peak amplitudes across repetitive stimulations (Fig. 3h). The baseline fluorescence intensity before the stimulations also remained stable throughout the imaging sessions (Supplementary Fig. 3). In contrast, slices loaded with non-anchored GINKO2 exhibited a gradual decrease in fluorescence intensity with repeated stimulation trials, indicating a time-dependent loss of indicator molecules over time because of diffusion. These findings suggest that, unlike the non-anchored form where only a small fraction of indicator molecules remain in the extracellular space, the lipid-PEG anchoring strategy stably retains the indicator on the plasma membrane, preventing diffusion-induced loss and enabling reliable detection of extracellular K⁺ dynamics over repeated stimulations.

### Semi-quantitative estimation of extracellular potassium ion concentration in mouse hippocampal slices along with the electrical stimulations

In general, single FP indicators report only relative values of molecular concentrations without offering absolute values. However, as we are monitoring molecules in extracellular spaces, we might be able to estimate absolution concentration of the molecules of interest by calibrating the indicators with standard solutions of known concentration of the molecules of interest. To estimate the absolute concentrations of extracellular K⁺ concentrations in intercellular spaces, after the recording of synaptic responses, we perfused the slices with aCSF containing known K⁺ concentrations and measured the fluorescence intensity changes.

Interestingly, the intrinsic fluorescence signals from unstained slices showed a decrease in their intensity in response to elevated extracellular K⁺ concentrations (Fig. 3i, gray). This finding suggests that estimating K⁺ concentrations solely from the fluorescence intensity using GINKO2 might be partially interfered with the contribution of intrinsic fluorescence signal change. Aside from this correction, we performed a semi-quantitative estimation based on averaged fluorescence values obtained from multiple brain slices.

As an initial step, we calculated the mean fluorescence intensity of unstained slices as a function of extracellular K⁺ concentration, to normalized the responses obtained from lipid-PEG-anchored GINKO2-stained slices over time and estimate fluorescence changes specifically attributable to the GINKO2 (Fig. 3i, blue). From these corrected fluorescence values, we generated a calibration curve using a Michaelis–Menten type four-parameter logistic function to normalize the indicator’s responsiveness to ligand concentration (Fig. 3j). The limit of detection (LOD) was 2.83 mM, and the range defined by the bend points of the four-parameter logistic curve fit^27^, was 6.03–222.68 mM. Using this calibration, the estimated baseline extracellular K⁺ concentration was approximately 2.5 mM, and the peak concentration in response to electrical stimulation (20 Hz, 5 pulses) reached 3.41 ± 0.45 mM. These estimated values were consistent with previous reports using K⁺-selective microelectrodes^28,29^.

Collectively, these findings demonstrate that lipid–PEG anchoring effectively retains FP-based indicators on the extracellular surface, enabling stable and reproducible monitoring of extracellular molecular dynamics near the cell membrane. Moreover, with appropriate calibration, this approach allows approximate quantification of extracellular ion concentrations in complex tissue environments.

### Imaging of stimulus-evoked responses of extracellular potassium ions and glutamate dynamics in mouse hippocampus slices

As we demonstrated that lipid-PEG-anchored GINKO2 can monitor extracellular K⁺ dynamics in acute mouse hippocampal slices, we next applied this method to observe glutamate release from neurons in acute hippocampus slices loaded with lipid-PEG anchored R-iGluSnFR1. While optically monitoring theindicator fluorescence, we simultaneously recorded field excitatory postsynaptic potentials (fEPSPs). Slices loaded with lipid-PEG-anchored indicator was stimulated at the Schaffer collateral with a stimulation electrode (50 or 100 μA, 20 Hz, 1–5 pulses).

**Fig. 4:**
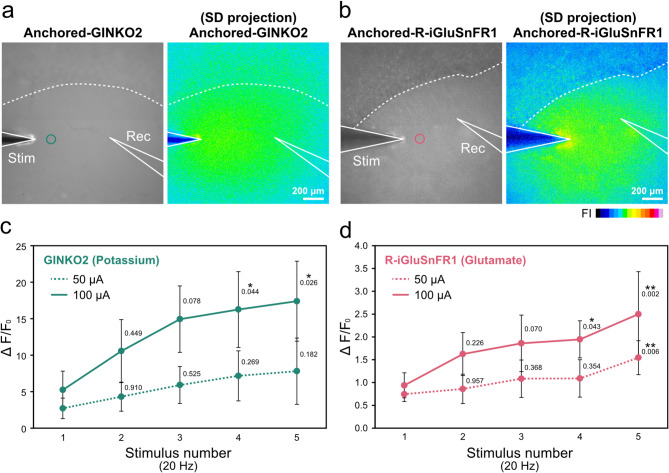
Relationship between field stimulation parameters, fluorescence responses, and fEPSP in acute hippocampal slices. (**a, b**) Fluorescence images of mouse hippocampal slices loaded with lipid-PEG-anchored GINKO2 (**a**) or lipid-PEG-anchored R-iGluSnFR1 (**b**), together with their time projection images. White lines indicate electrode positions, and dotted lines indicate the boundary of the CA1 pyramidal cell layer. Areas indicated by circles represent the regions for the fluorescence intensity measurements. Scale, 200 μm. (**c, d**) Comparison of fluorescence changes (ΔF/F_0_) of GINKO2 (**c**) or R-iGluSnFR1 (**d**) in response to 1–5 repetitive stimuli with an intensity of 50 μA (dashed line) or 100 μA (solid line). The values represent the means ± standard deviation (SD) (each sample; three independent slices). Statistical significance was assessed using one-way ANOVA with Dunnett’s multiple comparison test (∗ *p* < 0.05; ∗  ∗ *p* < 0.01; ∗  ∗  ∗ *p* < 0.001). *p*-values are indicated next to the SD bars.

Observation of R-iGluSnFR1 fluorescence changes from hippocampus slices after the electrical stimulations revealed that the dominant changes were observed mainly in the stratum radiatum, while the changes at the pyramidal cell layer (Fig. 4a) were relatively smaller than those observed in the stratum radiatum. In contrast, GINKO2 signals were observed not only in the stratum radiatum but beyond the pyramidal cell layer into the stratum oriens (Fig. 4b). Although differences in the spatial spread of K⁺ and glutamate signals could, in principle, be influenced by variations in tissue penetration or adsorption properties of the indicators, such effects are unlikely to be substantial. This is because the physicochemical properties of the lipid–PEG anchor and the molecular sizes of the FP-based indicators are comparable, making large differences in tissue accessibility between indicators improbable. In the stratum oriens, extracellular glutamate concentrations may be slightly elevated, consistent with previous reports describing layer-specific differences in astrocyte distribution and morphology^30^.

Field excitatory post synaptic potentials (fEPSPs) reflect the population activity elicited by electrical stimulation (Supplementary Fig. 4a, b), especially the initial slope of fEPSPs reflects fast synaptic transmission via non-NMDA components^31^. Increasing stimulus number and intensity caused increases in GINKO2 and R-iGluSnFR1 fluorescent signals (Fig. 4c, d, Supplementary Fig.2c-f). These changes suggest that electrical stimulation-evoked synaptic signaling induces glutamate releases, which in turn drives elevated extracellular potassium levels through depolarization-dependent mechanisms. Notably, GINKO2 responses approached a plateau upon the repeated stimulation (20 Hz) at 100 μA, suggesting the saturation of extracellular K^+^ concentration near the stimulation electrode.

These observations are consistent with the following sequence of events: electrical stimulation of the Schaffer collateral induces depolarization of axons. This is followed by glutamate release from presynaptic terminals, activation of postsynaptic glutamate receptors, depolarization and action potential generation by pyramidal neurons. K⁺ efflux occurs both at presynaptic and postsynaptic neurons along with the depolarizations^32^. Under physiological neuronal activity, extracellular K⁺ concentration change is thought to be caused by voltage-gated K⁺ channels activated during action potentials and are further shaped by synergistic contributions from multiple pathways, including postsynaptic NMDA receptors (NMDARs), AMPA receptors (AMPARs), Ca^2^⁺-dependent K⁺ channels and potassium ion transporters^33–35^.

### Pharmacological study of extracellular glutamate dynamics in mouse hippocampus slices

To further understand how glutamate diffuses into extracellular space using anchoring R-iGluSnFR1 in this approach, we inhibited glutamate uptake and monitored the resulting changes in fluorescence signals. DL-threo-β-Benzyloxyaspartic acid (TBOA), a competitive and non-transportable inhibitor of excitatory amino acid transporters (EAATs)^36,37^, was applied to mouse hippocampal slices loaded with lipid-PEG-anchored R-iGluSnFR1, while performing simultaneous fEPSP recordings and fluorescence imaging.

**Fig. 5:**
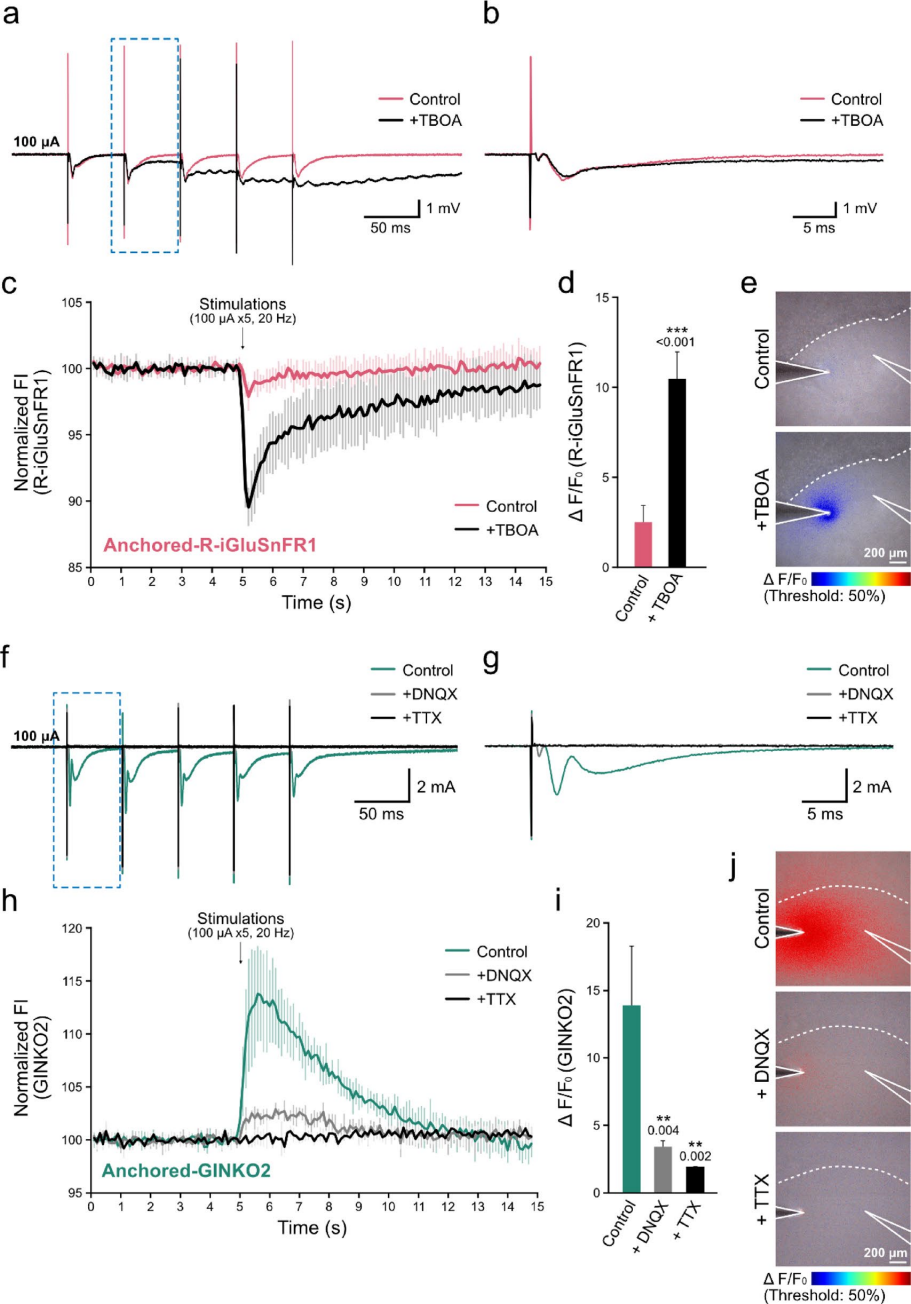
Pharmacological study of field stimulation–evoked fluorescence responses in acute hippocampal slices. (**a, b**) Representative fEPSP traces in the presence of 50 μM TBOA, evoked by five repetitive stimuli (100 μA, 20 Hz, 5 pulse) (**a**), and a magnified waveform of one response (**b**). (**c**) Time course of fluorescence intensity (FI) in slices loaded with lipid-PEG-anchored R-iGluSnFR1 under 50 μM TBOA. FI was normalized to the mean over 3 s pre-stimulation average (set as 100%). (**d**) Comparison of peak FI values from (**c**). Values represent means ± SD of five independent slices. Statistical significance was assessed using Welch’s *t* test (∗ ∗  ∗ *p* < 0.001). *p*-values are indicated above the SD bars. (**e**) FI mapping of lipid-PEG-anchored R-iGluSnFR1. Highlighted regions indicate pixels that exhibited > 50% change from baseline, as shown in (**c**). White lines indicate electrode positions, and dotted lines indicate the boundary of the CA1 pyramidal cell layer. Scale, 200 μm. (**f, g**) Representative traces of fEPSP responses in the presence of 50 μM DNQX or 1 μM TTX, evoked by repetitive electrical stimuli (100 μA, 20 Hz, × 5) (**f**), and one of their magnified waveforms (**g**). (**h**) Time courses of fluorescence intensity in slices loaded with lipid-PEG-anchored GINKO2 in the presence of 50 μM DNQX or 1 μM TTX. FI was normalized to the mean over 3 s pre-stimulation average (set as 100%). (**i**) Comparison of peak fluorescence intensity values in (**h**). The values represent means ± SD (each sample; three independent slices). Statistical significance was assessed using one-way ANOVA with Dunnett’s multiple comparison test (∗ ∗ *p* < 0.01). *p*-values are indicated above the SD bars. (**j**) FI mapping of lipid-PEG-anchored GINKO2. Highlighted regions indicate pixels that exhibited > 50% change from baseline, as shown in (**h**). White lines indicate electrode positions, and dotted lines indicate the boundary of the CA1 pyramidal cell layer. Scale, 200 μm.

Application of TBOA exerted very limited influence on the initial slope of the fEPSP evoked by electrical stimulation (Fig. 5a, b). This result indicates that presynaptic glutamate release remained largely unaltered and that the initial AMPAR-mediated postsynaptic response was unaffected. In contrast, TBOA markedly prolonged its decay phase. This is consistent with previous reports suggesting that glutamate persisted within the synaptic cleft for an extended period, leading to sustained activation of receptors such as NMDARs^38,39^. Repetitive stimulation further accumulated this activity-dependent effect, abolishing recovery to baseline and inducing a prolonged excitatory response. Consistent with these electrophysiological observations, the fluorescence intensity change (ΔF/F₀) of R-iGluSnFR1 increased significantly in the presence of TBOA compared to the control condition following electrical stimulation (100 μA, 20 Hz, 5 pulses) (Fig. 5c, d). This result indicates that TBOA suppressed glutamate uptake, leading to enhanced accumulation of extracellular glutamate and prolonged receptor activation, and further suggest that lipid-PEG-anchored R-iGluSnFR1 captures extracellular glutamate at the surface of living cell membranes.

Next, to assess the extracellular spread of glutamate, time-lapse fluorescence images were analyzed on a pixel-by-pixel basis. For each pixel, baseline fluorescence intensity (F₀, 0 s) and post-stimulation fluorescence intensity (F) were obtained, and the relative fluorescence change (ΔF/F₀) was calculated. Pixels exhibiting ΔF/F₀ values greater than 50% were extracted and mapped as representative regions of pronounced glutamate transients. Interestingly, after TBOA treatment, the area of R-iGluSnFR1 fluorescence changes were confined within approximately 100–200 µm from the tip of stimulating electrode and along the Schaffer collateral axonal projections (Fig. 5e). Extracellular glutamate released from neurons is known to be rapidly cleared by astrocytic transporters EAAT1/GLAST and EAAT2/GLT-1^40,41^. Taken together, these observations suggest that the regions exhibiting stimulus-dependent increase in extracellular glutamate are confined to a restricted area surrounding the stimulating electrode, corresponding to the region of active synaptic transmission.

### Pharmacological study of extracellular potassium ion dynamics in mouse hippocampus slices

Extracellular glutamate is known to activate AMPARs, leading to postsynaptic depolarization, followed by Na⁺ and Ca^2^⁺ influx and K⁺ efflux. To further examine the dynamics of extracellular K⁺, we applied lipid-PEG-anchored GINKO2 to mouse hippocampal slices and monitored fluorescence while simultaneously recording fEPSPs under pharmacological manipulations. Application of the AMPA- and kainate- receptor antagonist 6,7-dinitroquinoxaline-2,3-dione (DNQX) significantly reduced stimulus-evoked fEPSP amplitudes (Fig. 5f, g). In the presence of DNQX, the maximal fluorescence change of GINKO2 was also significantly decreased (Fig. 5h, i). Based on the calibration described above, the extracellular K⁺ concentration change evoked by electrical stimulation (100 μA, 20 Hz, 5 pulses) was estimated to increase by 4.25 ± 1.52 mM in control slices, while in the presence of DNQX the increase was reduced to 1.08 ± 0.12 mM, corresponding to an approximate 75% reduction of the condition without DNQX. These results indicate that the majority of extracellular K⁺ originated from postsynaptic cells. The residual K⁺ release in the presence of DNQX may originate from neurons directly depolarized by the stimulating electrode via action potentials.

To test this possibility, we applied tetrodotoxin (TTX), a voltage-gated Na⁺ channel blocker. Under TTX treatment, fEPSPs were completely abolished (Fig. 5f, g), and stimulation-induced GINKO2 fluorescence changes were negligible (Fig. 5h, i), corresponding to an estimated extracellular K⁺ increase of 0.69 ± 0.01 mM. We estimate that extracellular K⁺ release by electrical stimulation comprises approximately 75% from synaptically activated neurons, 9% from presynaptic neurons activated by voltage-gated Na⁺ channel, and 16% from other sources, such as direct depolarization of pyramidal cells by the stimulus. Thus, the majority of extracellular K⁺ arises from postsynaptic neurons, while directly stimulated neurons contribute only a minor fraction.

We also analyzed the spatial distribution of GINKO2 fluorescence changes. Because the indicator is localized exclusively to the cell surface, this approach allows high-sensitivity spatial detection of extracellular signals. Using the same approach described above to map the glutamate transients, we visualized the spatial distribution of GINKO2 fluorescence changes to assess the extracellular spread of potassium ions. Interestingly, the increase in GINKO2 fluorescence signals extended approximately 500 μm further from the tip of stimulating electrode spread into the CA1 pyramidal cell layer (Fig. 5j). These observations suggest that extracellular K⁺ increase spread over a broad area, potentially modulating extensive neuronal activity.

In this study, we demonstrate that the spatial distribution of extracellular ionic signals can be captured as fluorescence images, enabling visualization at both cellular and regional scales. Unlike point-based approaches such as microelectrodes or transistor-based sensors, fluorescence imaging intrinsically provides spatial information that cannot be obtained from localized measurements alone. Therefore, this optical readout offers enhanced spatial resolution for mapping ion dynamics across tissue regions.

## Conclusion

In this study, we showed that lipid-PEG anchoring of FP-based indicators enables imaging of extracellular molecular dynamics in live cells and living tissues. Importantly, this lipid-PEG anchoring method allows extracellular imaging of FP-based indicators even when the genetic expression to cell surface is difficult, thereby ensuring the application of FP-based indicators for broader range of biological activity not only in the cytoplasm but the extracellular space. As the reporter molecules are applied externally, this method enables immediate application of imaging extracellular space without genetic manipulation, which facilitates its use of FP-based indicators regardless of the difficulty in the genetical manipulation, such as the case for acute slice preparations. In addition, the use of homogeneous, purified sensor proteins ensures reproducibility of the experiments as the sensitivity and the stability of the sensor proteins can be tested separately. Collectively, the lipid-PEG anchoring strategy provides a versatile means of uniformly presenting sensor proteins on the plasma membranes of target tissues, potentially enabling high-spatial–temporal-resolution live imaging of extracellular molecular dynamics.

## Material and methods

### Materials

The following reagents were purchased: phosphate-buffered saline (PBS; FUJIFILM Wako Pure Chemical Corporation, Osaka, Japan), SUNBRIGHT OE-040CS (NOF CORPORATION, Tokyo, Japan), Dulbecco’s Modified Eagle Medium (DMEM; FUJIFILM Wako Pure Chemical Corporation), fetal bovine serum (FBS; Thermo Scientific), Oregon Green™ 488 BAPTA-1 (Invitrogen, Waltham, MT, USA), DL-threo-β-Benzyloxyaspartic acid (DL-TBOA; Funakoshi Co., Ltd., Tokyo, Japan), 6,7-Dinitroquinoxaline-2,3-dione (DNQX; FUJIFILM Wako Pure Chemical Corporation), and tetrodotoxin (TTX; FUJIFILM Wako Pure Chemical Corporation).

### Plasmid construction

GINKO2 and R-iGluSnFR were obtained from Addgene (#177,116 and #107,335, respectively). The indicator coding sequences were subcloned into the pRSET-A vector (Invitrogen, Waltham, MA, USA). For mammalian expression, the indicator sequences were further subcloned into pDisplay vector (Thermo Fisher Scientific, Walthman, MA, U.S.A.) or pcDNA3.1(-) vectors (Invitrogen). For membrane anchoring, PDGFR- and GPI-anchor sequences were fused to the N-terminus of the indicators. The PDGFR-anchor construct contained the IgK signal peptide (METDTLLLWVLLLWVPGSTGD) followed by the PDGFR transmembrane domain (AVGQDTQEVIVVPHSLPFKVVVISAILALVVLTIISLIILIMLWQKKPR), while the GPI-anchor construct contained the IgK signal peptide (METDTLLLWVLLLWVPGSTGD) followed by the GPI signal sequence (LENGGTSLSEKTVLLLVTPFLAAAWSLHP).

### Protein expression

For protein expression, *Escherichia coli* JM109 (DE3; Promega, Madison, WI, U.S.A.) was transformed with GINKO2 or R-iGluSnFR1 in the pRSET-A vector, and cultured in 200 mL LB medium with 50 μg/mL ampicillin (FUJIFILM Wako Pure Chemical Corporation, Osaka, Japan) at 20 °C for 3 days, and harvested by centrifugation. The harvested cells were suspended in phosphate-buffered saline, PBS( −) (pH 7.3), and lysed by three freeze–thaw cycles and sonication with 40 μg/mL lysozyme (FUJIFILM Wako Pure Chemical Corporation). After centrifugation, the supernatants containing the sensor protein were collected and purified on a Ni–NTA agarose column (QIAGEN, Venlo, Netherlands) followed by purification through a PD-10 gel filtration column (GE Healthcare, Buckinghamshire, U.K.) to remove imidazole and elution in PBS(-). The purified proteins were dispensed and stored at − 80 °C until further use.

### Lipid-PEG modification of indicators

Lipid-PEG modification was performed as previously described^20^. Briefly, 75 μM of purified indicator in PBS (95 μL) was mixed with 15 mM SUNBRIGHT OE-040CS in DMSO (5 μL) and incubated at room temperature for 1 h. The reaction was then quenched by adding 1 M Tris–HCl (pH 8.0, 5 μL), and the modified indicator was stored at 4 °C until use.

### In vitro spectroscopy

Fluorescence spectra of purified proteins were measured using a fluorescence spectrophotometer (RF-5300PC, Shimadzu Corporation, Kyoto, Japan). GINKO2 was excited at 480 nm, and emission spectra were recorded from 500–600 nm in the presence or absence of 100 mM K⁺ (peak near 515 nm). The emission wavelength set to 540 nm, excitation spectra in the range of 450–515 nm were recorded in the presence or absence of 100 mM K⁺. R-iGluSnFR1 was excited at 540 nm, and emission spectra were recorded from 550–650 nm in the presence or absence of 10 μM glutamate (peak near 585 nm).

Dose–response curves for purified GINKO2 and lipid-PEG–modified GINKO2 were obtained by measuring fluorescence intensity in solutions containing varying concentrations of K⁺ (0–100 mM). Similarly, dose–response curves for purified R-iGluSnFR1 and lipid-PEG–modified R-iGluSnFR1 were obtained using varying concentrations of glutamate (0–10 μM). EC₅₀ values were calculated using the Curve Fitter function in Rodbard mode of ImageJ (National Institutes of Health, Bethesda, MD, USA) by fitting fluorescence intensity data to a four-parameter logistic equation.

### Animal ethics and experimental procedures

All experimental procedures involving animals were conducted in strict accordance with the relevant guidelines and regulations. All protocols were reviewed and approved by the National Institute of Advanced Industrial Science and Technology Animal Care and Use Committee under approval number 2024–0454, 2025–0454. ICR mice were purchase from CLEA Japan, inc (Tokyo, Japan) and housed under controlled temperature and humidity with a 12-h light/dark cycle with food and water provided ad libitum. Euthanasia was performed under deep isoflurane anesthesia (5%) either by decapitation for the preparation of primary neurons or by exsanguination for acute slice preparation, in accordance with the AVMA Guidelines for the Euthanasia of Animals (2020) and institutional standard operating procedures. All procedures were carried out by trained personnel using appropriate, well-maintained equipment to minimize pain and distress. All experimental procedures are reported in accordance with the ARRIVE guidelines (https://arriveguidelines.org) ^42^.

### Cell Culture

HEK293 cells were cultured in high-glucose DMEM supplemented with 2 mM L-glutamine, 1 mM sodium pyruvate, 10% (v/v) FBS, 100 U/mL penicillin, and 0.1 mg/mL streptomycin (nacalai tesque, Kyoto, Japan) at 37 °C in a humidified 5% CO₂ incubator. For imaging experiments, 1.0 × 10^5^ cells were seeded onto 35-mm glass-bottom dishes coated with 1 mg/mL poly-L-lysine (Sigma-Aldrich) and cultured for 2 days before the imaging experiments.

### Primary mouse hippocampus neurons

Primary hippocampus neurons were prepared from embryonic 14–15-day fetal mice. Hippocampi were treated with trypsin (0.25% for 20 min, Nacalai tesque, Kyoto, Japan) in Hank’s balanced solution and gently dissociated. The obtained neurons were plated on poly-ethylenimine (0.1%, Sigma) coated glass bottom dishes at a density of 2–5 × 10^4^ cells/dish. Neurons were cultured in MEM culture medium (Nissui, Tokyo, Japan), supplemented with L-alanyl-L-glutamine (0.5 mM, Nacalai tesque), fetal bovine serum (2%), NeuroBrew (2%, Miltenyi Biotec, Tokyo, Japan), HEPES (10 mM, pH7.4) and glucose (0.45%).

### Acute slice preparations

The slices were prepared as described previously^43^ with some modifications. Briefly, mice were deeply anesthetized with isoflurane vapor (5%) in a closed chamber until respiration slowed and subsequently euthanized by decapitation. Brains were gently and quickly isolated and chilled for ~ 5 min in ice-cold cutting aCSF, which contains 220 mM Sucrose, 1.25 mM NaH_2_PO_4_, 2.5 mM KCl, 24 mM NaHCO_3_, 10 mM glucose, 0.2 mM CaCl_2_, 2.3 mM MgCl_2_ and 1.3 mM MgSO_4_, saturated with 95%O_2_/5%CO_2_ (carbogen). The brain was glued on cutting-chamber and sliced (300 µm thickness) with vibratome (Dosaka EM, Kyoto, Japan). Each slice was transferred immediately to recover chamber with pre-warmed aCSF, which contains 125 mM NaCl, 1.25 mM NaH_2_PO_4_, 2.5 mM KCl, 24 mM NaHCO_3_, 10 mM glucose, 2.5 mM CaCl_2_ and 1.3 mM MgSO_4_, 34℃, for 30 min, and then kept at room temperature at least 1.5 h before use.

### Transfection, and Lipid-PEG anchor application

For transfection, 1.5 μg of the expression plasmid was mixed with 3 μL Lipofectamine 2000 (Life Technologies, Carlsbad, CA, USA) and added to the culture medium. After medium replacement, cells were incubated for an additional 2 days at 37 °C. For lipid-PEG anchor experiments, lipid-PEG was added at a final concentration of 1 μM (cultured cell) or 5 μM (acute slice) and incubated at room temperature for 15 min. For calcium imaging, Primary mouse hippocampus neurons were incubated with 0.16 μM Oregon Green BAPTA for 30 min. Prior to imaging, cells were washed with the recording buffer (124 mM NaCl, 4.4 mM KCl, 1.25 mM NaH_2_PO_4_, 1.3 mM MgSO_4_, 2.5 mM CaCl_2_, 25 mM Glucose, 10 mM HEPES-Na [pH 7.4]) and imaged.

### Live-cell imaging of HEK293 Cells

Live-cell imaging of HEK293 cells was performed using an inverted fluorescence microscope (IX-81, Olympus, Tokyo) equipped with a 40 × oil-immersion objective lens (UPlanFL N, NA 0.75, Olympus), an EM-CCD camera (Roper Scientific, Trenton, NJ, USA), and a xenon illumination lamp (Olympus). GINKO2 fluorescence was acquired using a filter set consisting of an excitation filter (460–495 nm), emission filter (510–550 nm), and dichroic mirror (505 nm) (U-MWIBA2, Olympus). R-iGluSnFR1 fluorescence was acquired with the same filter set (U-MWIBA2, Olympus). The objective lens, stage, and perfusion tubing were maintained at 37 °C. Images were acquired every 5 s using µManager software (an open-source software program)^44^. Prior to imaging, cells were washed with the imaging buffer (140 mM NaCl, 3.5 mM KCl, 0.5 mM NaH_2_PO_4_, 0.5 mM MgSO_4_, 1.5 mM CaCl_2_, 10 mM HEPES, and 2 mM NaHCO_3_ [pH 7.4]). Cells were perfused with imaging solution (low potassium ion) or imaging solution with high potassium ion (93.5 mM NaCl, 50 mM KCl, 0.5 mM NaH_2_PO_4_, 0.5 mM MgSO_4_, 1.5 mM CaCl_2_, 10 mM HEPES, and 2 mM NaHCO_3_ [pH 7.4]) or imaging solution containing 1 mM glutamate during image acquisition.

### Live-cell imaging of primary neurons

Primary cultured neuronal cells on coverslips were observed with a Nikon Ti2-E microscope equipped with a 100 × Plan Apo TIRF objective (NA 1.49). A 20 mW, 488 nm and a 35 mW, 561 nm diode-pumped solid-state lasers (Melles Griot Optical Systems, Tokyo, Japan) provided illumination, introduced through an optical path installed on a vibration-insulated table (Herz). The setup included neutral density filters and an electromagnetic shutter (Vincent Associates) in the optical path. Excitation was achieved using a TIRF-illumination system that ensured uniform excitation of fluorescent molecules. We employ a circularly polarized laser beam that is rotated in the back focal plane of the objective with rotation frequency higher than 300 Hz to achieve isotropic TIR illumination at the specimen plane. The beam was scanned along a circular annulus in the BFP using a pair of galvo-scanned mirrors (GVS202, Thorlabs). The radius of the annulus in the BFP was adjusted such that the collimated laser reached the specimen plane beyond the critical angle required for TIR. The resulting fluorescence was captured on a CMOS camera (ORCA Fusion, Hamamatsu Photonics).

### Live-cell imaging of acute slices

Confocal imaging of acute hippocampal slices was performed using a confocal fluorescence microscope unit (FLUOVIEW FV1000, Olumpus) equipped with a 10 × objective lens (UPlanSApo, NA 0.40, Olympus), a 40 × objective lens (UPlanSApo, NA 0.90, Olympus), or a 100 × oil-immersion objective lens (UPlanApo, NA 1.35, Olympus). Fluorescence indicators were excited using a 488 nm diode-pumped solid-state laser (Ar 488, Melles Griot Optical Systems).

For live imaging, hippocampal slice was placed on custom-made recording chamber, in which both sides of the slice were perfused with carbogenize aCSF at 2–3 mL/min. Field EPSPs were elicited by 10–100 μA constant currents pulse (100 μs duration) with Tungsten bipolar electrode (Harvard Apparatus, Holliston, MA, USA) onto the Schaffer collateral and recorded with glass electrode filled with aCSF (1–2 MΩ). Field EPSPs were recorded with multiclamp700B (Molecular Device, San Jose, CA, USA) and digitized at 10 Hz (National Instruments, USA) with pclamp10 (Molecular devices LLC, San Jose, CA, USA). Fluorescence images were captured using IX51WI upright microscope (Olympus, Tokyo, Japan) equipped with Orca-Flash CCD camera (Hamamatsu photonics, Hamamatsu, Japan) controlled by µManager software (an open-source software program)^44^ with a custom script to synchronize pclmap10 software.

### Quantitative and statistical analysis

Fluorescence intensities were quantified using ImageJ/Fiji software (National Institutes of Health, Bethesda, MD, U.S.A.). For dose–response and calibration curve analyses, the fluorescence data were fitted with a four-parameter logistic function using the Rodbard mode in the *Curve Fitting* plugin of ImageJ/Fiji. The limit of detection (LOD) was defined as the mean plus three times the standard deviation of fluorescence intensity values obtained from stain-free slices. The linear range of the four-parameter logistic curve was calculated by the second-order derivative method^27^. Photobleaching correction was performed by fitting the fluorescence intensity decay from frames 1–49 with an exponential function.

For visualization of the magnitude and spatial distribution of fluorescence changes in Fig. 4, the standard deviation along the z-axis of the fluorescence stack was calculated for each pixel coordinate (x, y) and projected into a 2D map (ImageJ/Fiji, *Z-project [standard deviation]*). For fluorescence intensity (FI) mapping in Fig. 5, analyses were performed using Jupyter Notebook (Python). The FI mapping image represents areas exhibiting ≥ 50% change from the stacks.

For statistical analyses, data are presented as mean ± standard deviation (SD). Comparisons between two groups were performed using Welch’s *t*-test (Fig. 5d), and comparisons among multiple groups were performed using one-way ANOVA followed by Tukey’s post hoc multiple comparison test (Fig. 3e and S1b) or with Dunnett’s multiple comparison test (Figs. 4e, f and 5i) (*KyPlot 6.0*, KyensLab Inc., Tokyo, Japan).

## Supplementary Information

Below is the link to the electronic supplementary material.

Supplementary Material 1

Supplementary Material 2

Supplementary Material 3

## Data Availability

Raw imaging datasets are available from the corresponding author upon reasonable request.
