# Supplementary material for: Imaging intercellular biomolecules by using fluorescent protein indicators with lipid-PEG anchors
Source: Sci Rep. 2026 Feb 2;16:6964. doi: 10.1038/s41598-026-37240-4 (PMC12917146; doi:10.1038/s41598-026-37240-4)
Supplement: Supplementary file 3 — Supplementary Material 3 [file 41598_2026_37240_MOESM3_ESM.pdf]

Supplementary Information

Imaging Intercellular Biomolecules by Using Fluorescent Protein Indicators with Lipid-PEG Anchors

Marie Mita*, Kazuyuki Kiyosue, Tomomi Tani

Molecular Biosystems Research Institute, National Institute of Advanced Industrial Science and Technology (AIST), 1-8-31 B1-01111, Midorigaoka, Ikeda, Osaka, 563-8577, Japan

*Marie Mita, Ph.D. E-Mail: mita.marie@aist.go.jp

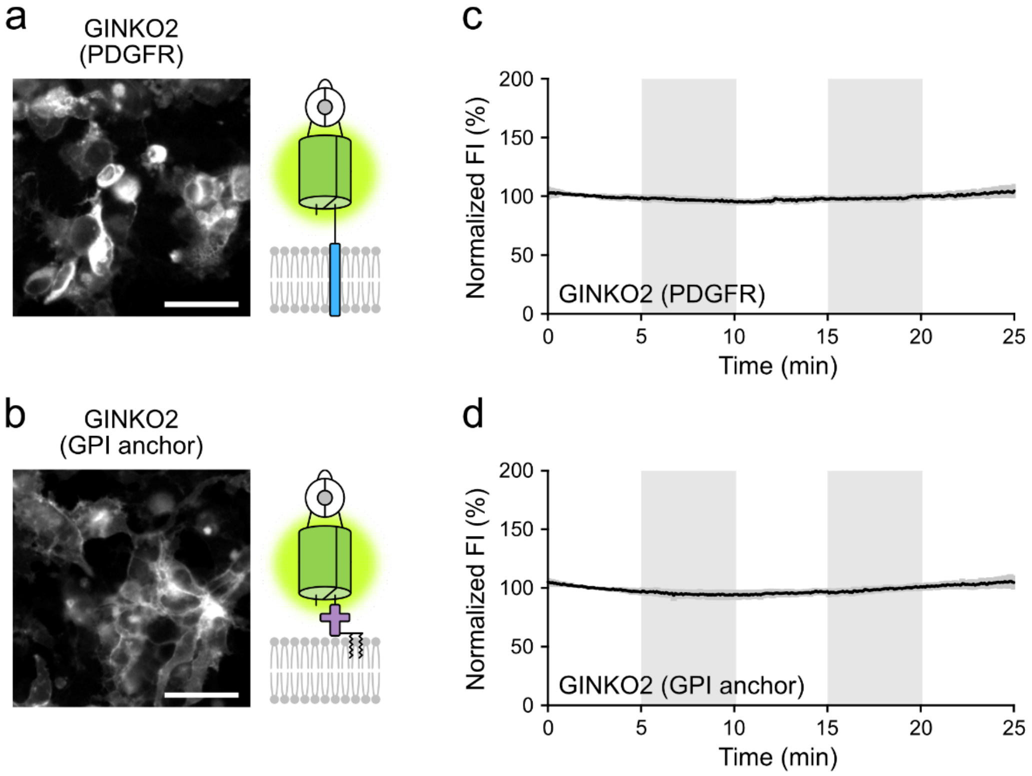

Supplementary Figure 1. Comparison of the genetical methods to expose fluorescent protein-based potassium indicators to the outer surface of HEK293 cell membrane. (**a**, **b**) Fluorescence images and schematic representations of fluorescent protein-based potassium ion indicator GINKO2 fused with a PDGFR transmembrane domain (**a**), a GPI anchor sequence (**b**). Scale: 50 μm. (**c**, **d**) Fluorescence intensity (FI) changes in HEK 293 cells expressing GINKO2 (PDGFR, **c**), GINKO2 (GPI anchor, **d**). Cells were perfused with solutions containing 3.6 mM K^+ (baseline) or 50 mM K^+ using a perfusion device. The solutions containing 50 mM K^+ was applied during the period indicated with gray backgrounds. FI values were normalized to the average FI over 5 min before the first stimulation (set as 100%). The values represent means ± SD of normalized fluorescence intensities (PDGFR, n = 30; GPI anchor, n = 40 cells from three or four independent experiments).

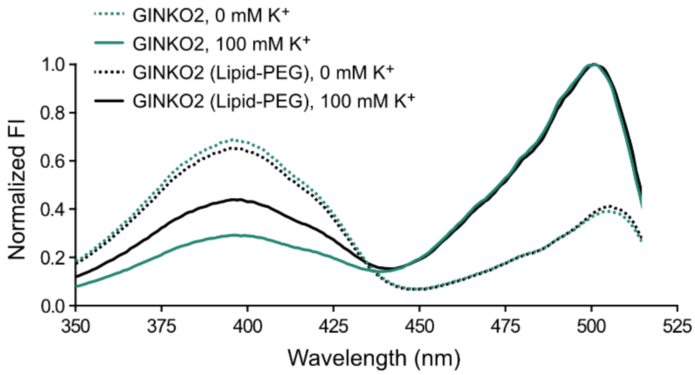

Supplementary Figure 2. Spectral property of GINKO2. Emission spectra of purified GINKO2 and GINKO2 lipid-PEG in the presence (solid line) or absence (dashed line) of 100 mM K^+. FI values were normalized to the maximal fluorescence intensity change observed in the presence of K^+.

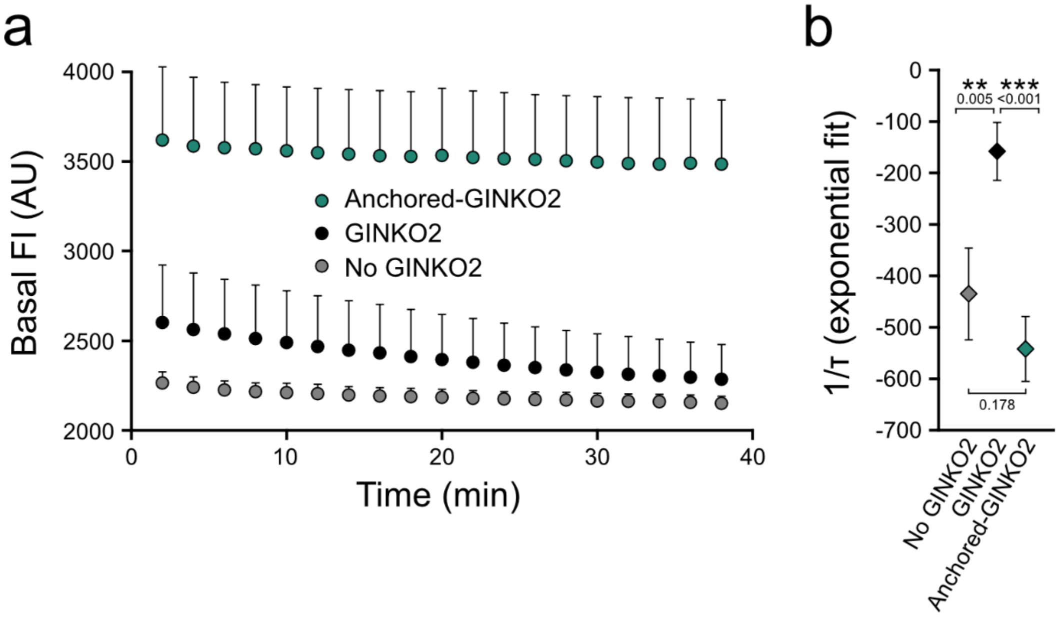

Supplementary Figure 3. Baseline fluorescence intensity changes in mouse hippocampus slices loaded with the lipid-PEG anchored GINKO2. (a) Baseline fluorescence intensity (FI) changes during repeated imaging in unstained (gray), non-anchored (black), and anchored slices (green). Values represent means ± SD (three independent slices). **(b)** Comparison of fluorescence decay rates fitted with an exponential function (y = a·exp^(τx)). Data represent means ± SD. Statistical significance was assessed using one-way ANOVA with Tukey's post hoc multiple comparison test (∗∗*p* < 0.01; ∗∗∗*p* < 0.001).

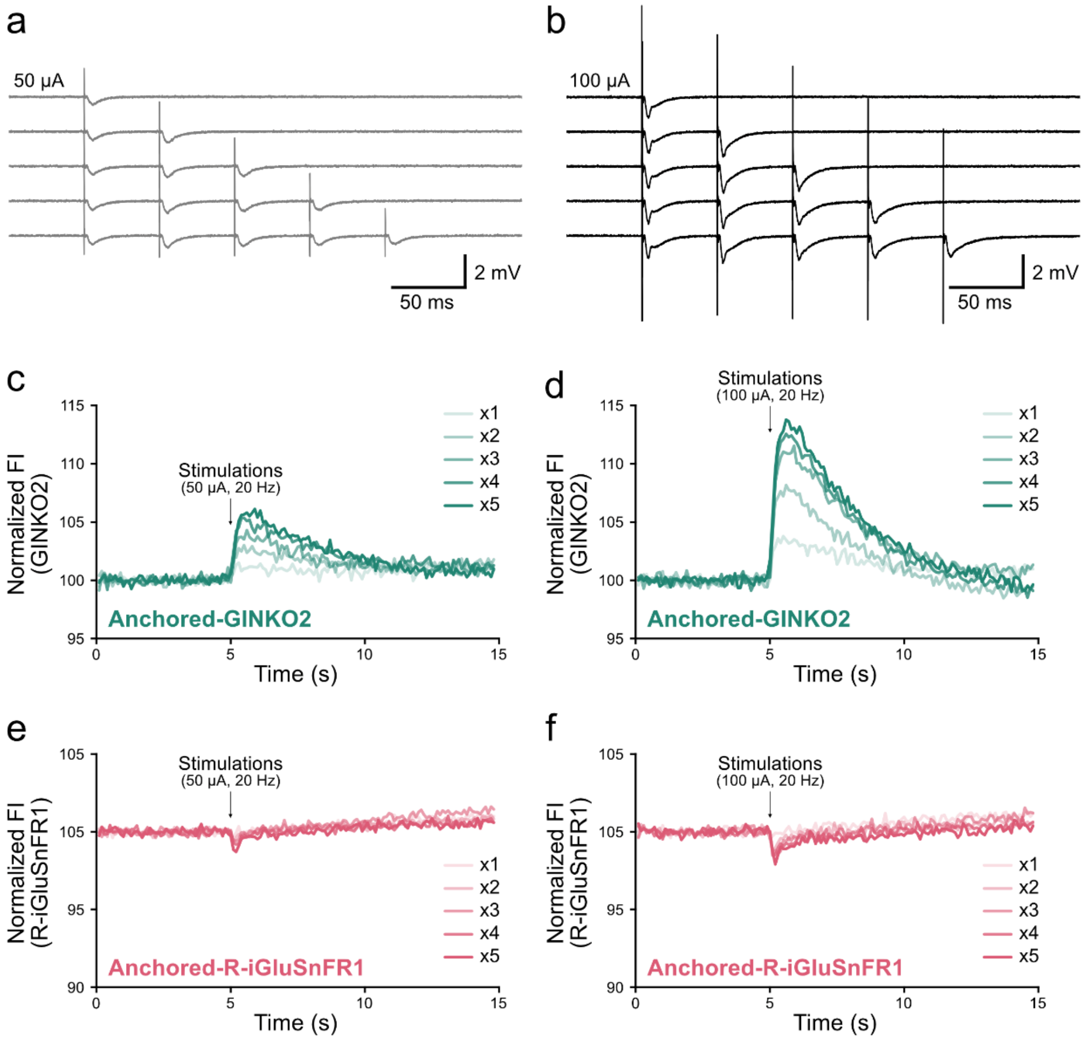

Supplementary Figure 4. Effects of varying field stimulation parameters on fluorescence responses and fEPSPs in acute hippocampal slices. (**a**, **b**) Representative traces of fEPSP responses evoked by 1-5 repetitive stimuli with an intensity of 50 µA (**a**) or 100 µA (**b**). (c, d) Time courses of fluorescence intensity changes during 1-5 pulses of electrical stimulation (20 Hz) at 50 µA (**c**) or 100 µA (**d**) in slices loaded with lipid-PEG-anchored GINKO2. Fluorescence intensity (FI) values were normalized to the 5 sec pre-stimulation average (set as 100%). Values represent means (three independent slices). (**e**, **f**) Time courses of fluorescence intensity changes during 1-5 pulses of electrical stimulation (20 Hz) at 50 µA (**e**) or 100 µA (**f**) in slices loaded with lipid-PEG-anchored R-iGluSnFR1. FI values were normalized to the 5 sec pre-stimulation average (set as 100%). Values represent means (three independent slices).

Supplementary video 1. Fluorescence intensity changes in HEK293 cells loading of lipid-PEG anchored GINKO2. HEK293 cells external loading of lipid-PEG anchored GINKO2, related in Fig. 1c and 1f. Cells were perfused with solutions containing 3.6 mM K^+ (baseline) or 50 mM K^+ using a perfusion device. The solutions containing 50 mM K^+ was applied during the period indicated with white circles. Scale: 50 µm.

Supplementary video 2. Fluorescence intensity changes in acute hippocampal slices loading of lipid-PEG anchored GINKO2. Fluorescence images (left) and corresponding fluorescence intensity traces (right) from acute hippocampal slices loaded with lipid-PEG-anchored GINKO2, corresponding to the control condition in Fig. 3d and 3f. The slice evoked by repetitive electrical stimuli (20 Hz, 5 pulse) indicated with white circles (5.0 sec). Scale: 200 µm.
